# Open-access publications: a double-edged sword for critical care researchers in lowand middle-income countries

**DOI:** 10.5935/2965-2774.20230263-en

**Published:** 2023

**Authors:** Antonio Paulo Nassar Jr, Flávia Ribeiro Machado, Felipe Dal-Pizzol, Jorge Ibrain Figueira Salluh

**Affiliations:** 1 Intensive Care Unit, A. C. Camargo Cancer Center - São Paulo (SP), Brazil; 2 Intensive Care Department, Hospital São Paulo, Escola Paulista de Medicina, Universidade Federal de São Paulo - São Paulo (SP), Brazil; 3 Laboratory of Experimental Pathophysiology, Postgraduate Program in Health Sciences, Health Sciences Unit, Universidade do Extremo Sul Catarinense - Criciúma (SC), Brazil; 4 Intensive Care Department, Instituto D’Or de Pesquisa e Ensino - Rio de Janeiro (RJ), Brazil

In recent decades, the landscape of scientific publications has dramatically changed with the introduction of the so-called open access (OA) model. In brief, a publication can be considered open access when there are no financial barriers to its access in a digital format. Although the initial moves in this chess game were met with skepticism by both publishers and scientists alike, OA publishing quickly became a standard model that encompassed both new and traditional, nonindexed and high-impact journals. In critical care, 3 out of the top 10 impact factor journals exclusively dedicated to the field are OA, and most of the others have gradually adhered to a partial OA model (where the authors may opt-in to an OA publication).

The data demonstrate that publications available as OA are highly cited, and this may be because some of the world’s most important funding agencies, such as the National Institutes of Health (USA), mandate that studies that are financed by them be published as OA. It is only fair to consider that OA publications have democratized access to science broadly, particularly in lowand middle-income countries (LMICs), where access is rather limited even in academic centers. However, when considering the perspective of LMICs or the “Global South” on OA in critical care, one piece of the puzzle remains missing: the researchers.

Currently, almost 86% of the world’s population lives in LMICs; therefore, research that is relevant and applicable to these countries is of paramount importance. During the COVID-19 pandemic, access to timely and reliable scientific information was crucial, but the ability to produce high-quality research was also a major part of the pandemic response that mitigated the catastrophic effects of its burden on ICUs. For LMIC-based critical care researchers, the winding road to scientific publication has many challenges, and one of them is the restricted access to funding, including funding for the publication of results.

## Open access model and lowand middle-income countries

Open access journals usually exempt researchers from low-income countries from publication fees, and this exemption seems to favor the publication of articles by researchers from sub-Saharan Africa.^(1)^ However, producing high-quality scientific research in these settings is challenging and often funded by agencies such as the National Institutes of Health (NIH), Wellcome and the Gates Foundation; consequently, the impact of exempting article processing charges (APCs) on publishers’ financial health is minimal. On the other hand, researchers from middle-income countries do not receive exemptions from APCs for publishing OA articles. Between 1996 and 2022, of the top 20 countries in number of publications, five were in this income category (China, India, Russia, Brazil, and Turkey) and accounted for 27% of all publications, according to the SCImago portal.^(2)^ A study encompassing Elsevier journals has shown that APCs are a barrier to OA publication.^(3)^

**Figure 1:**
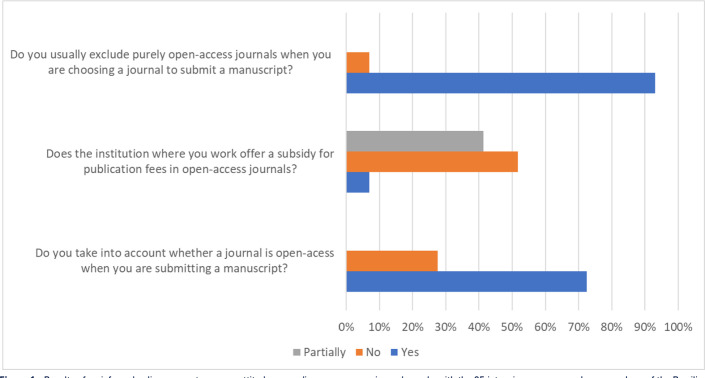
Results of an informal online survey to assess attitudes regarding open access journals made with the 35 intensive-care researchers, members of the Brazilian Research Intensive Care Network (BRICnet) in September 2023.

Three of the top 10 critical-care journals publishing exclusively in OA format (Critical Care, Annals of Intensive Care, and Journal of Intensive Care) had APC ranging from US$ 2,490 to US$ 3,790 in 2023. After correcting for purchasing power parity as calculated by the Organization for Economic Co-operation and Development (OECD) in 2022,^(4)^ these fees would correspond to the range from R$ 6.300 to R$ 9.600. These costs would correspond to 4.7 to 7.2 times the Brazilian minimum wage,^(5)^ but only 1.9 to 2.8 times the French minimum wage^(6)^ or 1.2 to 1.7 times the Canadian minimum wage in 2023.^(7)^ It is worth mentioning that Brazilian research grants do not earmark specific funds for paying the APCs of OA articles, an oversight that urgently needs to be acknowledged and changed. The NIH policy of mandating OA for the results derived from their grants is an example to be followed. An informal online survey of the 35 members of the Scientific Committee of the Brazilian Research in Intensive Care Network (BRICnet)^(8)^ in September 2023 to assess attitudes regarding OA journals received responses from 29 members. Broadly characterized, the vast majority of the researchers either excluded fee-based OA journals or took this journal feature into account when choosing a journal for submitting a manuscript, and researchers’ institutions either did not pay for publication fees or subsidized it only partially (Figure 1).

## Full open access: the Critical Care Science model

Critical Care Science is an official publication of the *Associação de Medicina Intensiva Brasileira* and the *Sociedade Portuguesa de Cuidados Intensivos*, OA and is indexed in PubMed®. Despite being OA, it has no APCs, meaning that there is no cost to the authors for publication. This model is only possible because these medical societies decided to fund the journal as a way to benefit the medical community. This model eliminates the publication barriers for both readers and those producing the science. Other leading societies in the field, by adopting similar policies, would highly contribute to the global improvement in quality of care and equity.

In the last 12 months, 71% of the submissions to Critical Care Science were from authors based in the “global south” (after excluding Brazil and Portugal from the list). What does this proportion mean? It clearly represents that the journal is diverse and publishes studies from the top countries producing science (i.e., USA, France, Canada), but it also means that our approach to full open access provides a well-paved road with no barriers for researchers based in LMICs.

Currently, the journal remains committed to its mission of remaining fully open access with no barriers for researchers and readers alike. This practice benefits all those interested and able to produce (and apply) novel scientific findings in critical care regardless of whether they are in highor lowor middle-income countries.

Open-access publications are essential to reduce the inequities of access to scientific publications. However, from the perspective of those producing science, publication remains relatively restrictive due to elevated article processing charges. Improving this model in terms of equity is the duty of academics, medical and scientific societies, funding agencies, and regulators if they aim to translate the rhetoric about “global health” into a reality that benefits everyone.
